# Mutations in the coding regions of the hepatocyte nuclear factor 4 alpha in Iranian families with maturity onset diabetes of the young

**DOI:** 10.1186/1475-2840-8-63

**Published:** 2009-12-10

**Authors:** Seyed Morteza Taghavi, Seyedeh Seddigheh Fatemi, Houshang Rafatpanah, Rashin Ganjali, Jalil Tavakolafshari, Narges Valizadeh

**Affiliations:** 1 Internal Medicine Department, Ghaem Hospital & Endocrine Research Center, Mashhad University of Medical Sciences, Parastar st, Ahmad abad blvd, Mashhad, Iran; 2 Immunogenetics Department, Immunology Research Center, Bu-Ali Research Center, Mashhad University of Medical Sciences, Bu Ali Square, Ferdowsi Square, Mashhad, Iran; 3 Microbiology and Virology Department, Bu-Ali Research Center, Mashhad University of Medical Sciences, Bu Ali Square, Ferdowsi Square, Mashhad, Iran

## Abstract

Hepatocyte nuclear factor 4α (HNF4α) is a nuclear receptor involved in glucose homeostasis and is required for normal β cell function. Mutations in the HNF4α gene are associated with maturity onset diabetes of the young type 1 (MODY1). The aim of the present study was to determine the prevalence and nature of mutations in HNF4α gene in Iranian patients with a clinical diagnosis of MODY and their family members. Twelve families including 30 patients with clinically MODY diagnosis and 21 members of their family were examined using PCR-RFLP method and in case of mutation confirmed by sequencing techniques. Fifty age and sex matched subjects with normal fasting blood sugar (FBS) and Glucose tolerance test (GTT) were constituted the control group and investigated in the similar pattern. Single mutation of V255M in the HNF4α gene was detected. This known mutation was found in 8 of 30 patients and 3 of 21 individuals in relatives. Fifty healthy control subjects did not show any mutation. Here, it is indicated that the prevalence of HNF4α mutation among Iranian patients with clinical MODY is considerable. This mutation was present in 26.6% of our patients, but nothing was found in control group. In the family members, 3 subjects with the age of ≤25 years old carried this mutation. Therefore, holding this mutation in this range of age could be a predisposing factor for developing diabetes in future.

## Background

Maturity onset diabetes of the young (MODY) is a genetically, metabolically, and clinically heterogeneous subtype of diabetes mellitus characterized by early onset autosomal dominant inheritance and beta cell dysfunction [1]. This monogenic disease accounts for 1-5% of all type 2 diabetes (T2D) cases and is characterized by high penetrance, early age at onset (usually before 25 years and often in adolescence or childhood), primary defect in insulin secretion, and mild to severe clinical manifestations [2]. Variants in six genes responsible for MODY have been identified: hepatocyte nuclear factor 4α (HNF4α/MODY1; MIM#600281) on chromosome 20q [3], glucokinase (GCK/MODY2; MIM#138079) on chromosome 7p [4], transcription factor 1 (TCF1/MODY3; MIM# 142410) on chromosome 12q [5], insulin promoter factor 1 (IPF1/MODY4; MIM#600733) on chromosome 13q [6], transcription factor 2 (TCF2/MODY5; MIM#189907) on chromosome 17q [7] and neurogenic differentiation factor 1 (NEUROD1/MODY6; MIM#601724) on chromosome 2q [8]. All of these genes encode proteins involved in the glucose homeostasis of the pancreatic β cell [9]. Heterozygous mutations in these genes appear to result in different clinical presentations. The recent identification of these MODY genes currently allows investigation of the specific defects present in each family and identification of the respective genotype-phenotype correlations [2]. Exact relative prevalence of distinct MODY subtypes is unknown and varies substantially in different populations [10-12]. Additionally, in several populations other yet unknown genes, known as MODY X may be responsible for up to 79% of MODY cases [13]. This finding suggests that additional MODY genes are likely to exist.

Hepatocyte nuclear factor 4α is a member of the steroid/thyroid hormone receptor super family and is expressed in the liver, kidney, small intestine and pancreatic islets [14,15]. It activates a wide variety of essential genes, including those involved in cholesterol, fatty acid and glucose metabolism and in liver differentiation [16]. The MODY1 phenotype is because of a loss of HNF4α function [17]. The molecular mechanisms by which cause mutations in HNF4α are not fully understood. Clinical studies suggest that MODY1 is characterized by a defect in glucose-stimulated insulin secretion, suggesting that abnormal gene expression in the pancreatic β cell is responsible for this disorder [18]. So, HNF4α is critical for regulating glucose transport and glycolysis and in doing is crucial for maintaining glucose homeostasis. Some missense mutations in the HNF4α gene have been shown to segregate with diabetes in MODY pedigrees in different populations.

The present study was undertaken to examine the prevalence and the nature of mutations in the HNF4α gene in a group of Iranian MODY patients and their family members, because identification of these mutations allowed for presymptomatic diagnosis in the younger generations and will improve medical follow up of the predisposed individuals. Diagnosis of MODY has important implications for clinical management.

## Subjects and Methods

Thirty patients with clinical feature of MODY were included in this study. The screening criteria we used were: 1) patient and at least one first degree relative with T2D diagnosed before 25 years of age, 2) laboratory approved hyperglycemia and clinical manifestations of diabetes, 3) positive family history for diabetes at least in two previous generations, and 4) entering the disease into the family on only one side. Patients with classic type 1 diabetes (acute ketotic presentation or continuous requirement of insulin within the first year of diagnosis) were excluded. Our patients were recruited from Endocrinology Clinic of Ghaem University Hospital in Mashhad, covering the general population of this city. The patients' families were screened further regarding the occurrence of T2D. Then they were asked to participate in our study. Fifty age and sex matched subjects which counted toward our control group were evaluated by FBS and GTT not to miss any undiagnosed diabetes.

This study was approved by ethics committee of Mashhad University of Medical Sciences. Written informed consent forms were filled for all the patients.

In the first part of the study, a medical questionnaire was filled for every patient including demographic data, the type of diabetes, duration of the disease, type of therapy whether to use insulin or oral hypoglycemic agents, duration and current dose of drug usage. Correspondently, height, weight and BMI were measured. Also information on the medical history of individual family members was obtained by another questionnaire.

In the latter step, blood samples were taken from the patients and their nominated relatives for DNA extraction and measurement of biochemical indices. Fasting plasma glucose, fasting serum insulin, and hemoglobin A_1C _were determined in all patients. Laboratory analyses were performed with commercially available standardized methods.

For screening of the mutations, genomic DNA was isolated from peripheral blood lymphocytes of patients and relatives by salting out method [19]. PCR- RFLP was carried out in all subjects took part in the study and additional process of sequencing was done in those cases in which a mutation was detected by PCR. The eleven exons and flanking introns in addition to Val/Met255 variant were amplified. Specific primer sequences are described in table 1. Our PCR mixture includes 200 ng of DNA, 10 pmoles of each primer, 200 μm dNTPs, 1.5 mM MgCl_2 _and Taq DNA polymerase (0.5 U/20 μl). The program of thermal cycler was 5 min denaturation at 94°C followed by 38 cycle of denaturation at 94°C for 1 min, annealing at T_anneal _(Table 1) for 1 min, and extension at 72°C for 1 min, with a final extension at 72°C for 5 min. Amplified restricted fragments were detected after digestion with BstBI, and AvaI was only used for Val/Met255 variant. The fragments were resolved on a 2% agarose gel and visualized by staining with ethidium bromide. PCR products were purified. For cycle sequencing, ABI (Applied Biosystems) BigDye 3.1 chemistry was used. Sequencing runs were performed on ABI 3730 sequencers with 50 cm capillaries. Double stranded sequencing was performed in cases we encountered difficulties in the interpretation.

**Table 1:** Nucleotide sequences of DNA primers used for PCR amplification of the HNF4α gene

Region	Sense primer (5'→3')		Antisense primer (5'→3')		Segment size	Tannealing/°C
1	tgtaaaacgacggccagtgggcactgggaggaggcagt		caggaaacagctatgacccttggcaacacctgtgctggc		405 bp	75

1b	tgtaaaacgacggccagttcatatcagcaacatgtccg		caggaaacagctatgaccgggctcttccctccagga		210 bp	73

2	tgtaaaacgacggccagtcttcctgaagcctcactcc		caggaaacagctatgacccccaagtgtgcccatttcc		352 bp	75

3	tgtaaaacgacggccagtgttgtgtcttctccatcca		caggaaacagctatgaccgcaggtggggcagtggtg		215 bp	56

4	tgtaaaacgacggccagttctccctcctcacctctctg		caggaaacagctatgacccctctgtagtgtggggga		226 bp	56

5	tgtaaaacgacggccagtatctccagcattttcttccc		caggaaacagctatgacccactgcccactactgccc		267 bp	72

6	tgtaaaacgacggccagtagggtacagatggcaaacac		caggaaacagctatgaccaccctccctggagccctg		204 bp	70

7	tgtaaaacgacggccagttgacttcccatcctccctcc		caggaaacagctatgaccggagagagagtcagggatgg		268 bp	68

8	tgtaaaacgacggccagtagctggaccctgctgccc		caggaaacagctatgacccactccaaccccgcccct		354 bp	74

9	tgtaaaacgacggccagtgcatcccagactctccatcc		caggaaacagctatgaccttgcaaggtaaaatcccagag		262 bp	72

10	tgtaaaacgacggccagtagcccctgtctgtctgtttg		caggaaacagctatgaccgggactggtcctggcatcac		316 bp	72

Val/Met255 variant	ccggagctggcggagatgacccg		caggaaacagctatgaccggagagagagtcagggatgg		180 bp	70

## Results

### Clinical and Biochemical features

A total of 12 families with an autosomal dominant pattern of occurrence of early onset diabetes were constituted the cohort of the study. Table 2 summarizes characteristics of the families. Most of the patients had an early onset of diabetes (mean onset age of 24). Selected clinical characteristics of the study subjects are demonstrated in table 3. We had 22 women and 8 men in patients group and 10 women and 11 men in the family members group. For comparison 50 sex and age matched healthy subjects were selected as control group included 30 women and 20 men with the range of age 25 to 35 years old. The diabetic patients, at the time of the study, had a body mass index (BMI) between 20 and 31 kg/m^2^. There was no significant difference between the patients' BMI and other two groups. So, obesity had no major impact on the clinical onset and course of diabetes hereby. At the time of diagnosis, all patients were being treated with oral hypoglycemic agents; however, 13.3% (4/30) of the patients were shifted to insulin therapy during the investigation. According to this point and the laboratory results; fasting blood glucose, fasting serum insulin HbA_1_c, and being well controlled patients, sensitivity of oral hypoglycemic agents in our MODY patients is noticeable.

**Table 2:** Characteristics of examined families

Family Number (Total Members)	Affected Subjects	Mean Age at Diagnosis (range)
1(2)	2	19(14-24)

2(3)	2	27(26-28)

3(7)	2	23.5(23-24)

4(5)	2	22(18-26)

5(6)	3	33(21-23)

6(5)	3	24(12-19)

7(6)	3	31.5(18-24)

8(2)	2	22.5(20-25)

9(2)	1	24

10(5)	4	50.5(21-33)

11(6)	4	52(23-30)

12(2)	2	24.5(24-25)

**Table 3:** Comparison of clinical characteristics between affected members, unaffected members of families with Maturity Onset Diabetes of the Young (MODY) and control subjects

	Affected Members	Unaffected Members	Control Subjects
Samples(n)	30	21	50

Male/Female	8/22	11/10	20/30

Age at Diagnosis(years)	24 ± 6	--------	--------

Age at Examination(years)	36 ± 5	56 ± 7	41 ± 6

BMI(kg/m^2^)	26.8 ± 9.2	25.2 ± 5.3	27.1 ± 4.2

Fasting Blood Glucose(mg/dl)	136 ± 62	--------	--------

Fasting Serum Insulin(μU/ml)	14.2 ± 5.4	--------	--------

HbA_1C_(%)	6.8 ± 2.1	--------	--------

### Mutation Identification

**Figure 1:**
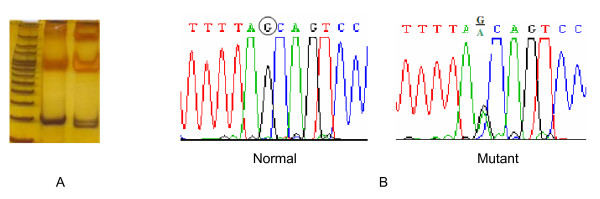
**(A): PCR-RFLP analysis of Val/Met255 variant**. (B): Partial sequence of selected region of HNF4α gene; Val/Met255 variant. The sequence of the normal and mutant alleles is shown. The circle indicates the G→A substitution at codon 255.

Comparison of sequencing results to reference gene sequence revealed a G→A substitution at codon 255 resulting in a Val (GTG) → Met (ATG) replacement (figure 1). This known mutation was identified in 8/30 (26.6%) of patients with MODY and 3/21 (14.2%) of relatives. No mutation was detected in healthy controls. The mean age of the patients at the time of diagnosis was 24 (12-30), whereas, these values were 21.5 (14-26) and 16 (12-19) in 8 cases with Val/Met255 mutation in patients group and 3 individuals with mutation in relatives, respectively.

## Discussion

HNF4α mutations have been shown to play role in an autosomal dominant manner in families with an atypical form of T2D known as maturity onset diabetes of the young (MODY1) [20,21]. HNF4α's role in MODY originates from its function as a β cell transcription factor that influences glucose induced insulin secretion [22]. The clinical features of the HNF4α phenotype as a subtype of MODY can overlap with type 1 diabetes. Occasionally, MODY patients may be misdiagnosed as having type 1 diabetes because they present with polydipsia, polyuria, and weight loss in their late teens or early 20s. In contrast to MODY, type 2 diabetes usually affects people between age 40 and 60 years, but an increasing number of patients are being diagnosed earlier, even in childhood. These patients are frequently obese and require insulin therapy. Approximately equal numbers of patients with MODY and T2D report a parent with diabetes and more than one out of three MODY patients can not be distinguished from those with T2D using traditional diagnostic criteria of age of onset and family history alone [23]. Both MODY and T2D patients, have reduced insulin sensitivity as a result of pancreatic β cell dysfunction [24]. So, MODY should be suspected in young onset apparent T2D cases when there is no obesity or feature of metabolic syndrome. In the case of encountering a patient with some manifestations of type 1 diabetes and some features of T2D, in addition to probable clinical diagnosis of MODY, genetic investigation of identified mutations could let to better diagnosis. This helps to predict the clinical course of disease and will influence the management. Finding mutations associated with MODY in probands has implications for other family members, particularly, identification of asymptomatic carriers that may be beneficial for earlier diagnosis and treatment in order to avoid complications. One of the genes to be investigated is HNF4α as a known cause of MODY1. The current study indicates the presence of Val/Met255 mutation in almost one forth of Iranian MODY. In one of the twelve families took part in this study, 3 individuals developed Val/Met255 mutation that were all under the age of 25 (12, 17 and 19 years old). Respecting the occurrence of this mutation in our MODY patients and probable development of these 3 members of this family to MODY, in future cases similar to these conditions, detection of this mutation may help the earlier diagnosis.

Approximately 5-10% of T2D cases are MODY. But Mutations in HNF4α (MODY1) are less frequent and may account for 2-5% of subjects with MODY [8,25,26]. Only 26 families worldwide have been diagnosed with MODY1 [9,27], although, we found HNF4α mutation (Val/Met255) in 3 families of our study group. This substitution changes an amino acid sequence of the HNF4α protein. In this study, none of the Val/Met255 carriers have overt hepatic, renal or gastrointestinal dysfunction. So, we believe this mutation might have an effect on the pancreatic beta cell function contributing to the development of MODY.

In summary, Val/Met255 mutation in the HNF4α gene was identified in about one fourth of Iranian families affected with MODY. Therefore, the detection of this mutation in an Iranian patient with probable MODY provides a strong support in the definite diagnosis. Although, we plan to investigate our patients and their family members to detect the common mutations of other subtypes of MODY in the future studies.

## Competing interests

The authors declare that they have no competing interests.

## Authors' contributions

SSF carried out the molecular genetic studies, participated in the sequence alignment and drafted the manuscript. SSF and RG carried out the immunoassays. HR and NV participated in the sequence alignment. SSF and SMT participated in the design of the study, visit and selection of the patients, and performing the statistical analysis. JT coordinated the study. All authors read and approved the final manuscript.
